# In vitro and in vivo targeted delivery of IL-10 interfering RNA by JC virus-like particles

**DOI:** 10.1186/1423-0127-17-51

**Published:** 2010-06-24

**Authors:** Meng-Ing Chou, Yu-Fan Hsieh, Meilin Wang, Jinghua Tsai Chang, Deching Chang, Moncef Zouali, Gregory J Tsay

**Affiliations:** 1 Institute of Medicine, Chung Shan Medical University, Taichung, Taiwan; 2 Institue of Immunology, Chung Shan Medical University, Taichung, Taiwan; 3 Department of Microbiology and Immunology, Chung Shan Medical University, Taichung, Taiwan; 4 Institute of Medical and Molecular Toxicology, Chung Shan Medical University, Taichung, 40201, Taiwan; 5 Department of Life Science, National Chung Cheng University, Chiayi County, Taiwan; 6 Institute National de la Santé et de la Recherche Médicale, INSERM U606, Centre Viggo Petersen, Hôpital Lariboisière, 2, Paris CEDEX 10, France; 7 University Denis Diderot Paris 7, 75475 Paris, France; 8 Department of Internal Medicine, Chung Shan Medical University Hospital, Taichung, Taiwan

## Abstract

**Background:**

RNA interference (RNAi) is a powerful tool to silence gene expression post-transcriptionally. Delivering sequences of RNAi *in vivo *remains a problem. The aim of this study was to use JC virus (JCV) virus-like particles (VLPs) as a vector for delivering RNAi in silencing the cytokine gene of IL-10.

**Methods:**

JCV VLPs were generated by recombinant JCV VP1 protein in yeast expression system. DNA fragment containing IL-10 shRNA was packaged into VLPs by osmotic shock.

**Results:**

In RAW 264.7 cells, IL-10 shRNA was found to reduce IL-10 expression by 85 to 89%, as compared with VLPs alone. IL-10 shRNA did not cross-react with TNF-alpha mRNA or influence the expression of TNF-alpha. In BALB/c mice IL-10 shRNA could reduce 95% of IL-10 secretion. Surprisingly, it also down regulated TNF-alpha expression.

**Conclusions:**

We show for the first time that JCV VLPs empty capsids are competent vectors to deliver RNAi and are nontoxic to cells, suggesting that JCV VLPs is an efficient agent to deliver RNAi in both murine macrophage cells and BALB/c mice. This system provides an efficient means for delivering the RNAi for gene therapy purposes.

## Background

Transfection of RNA interference (RNAi) into living cells is a major technique in studying the biological function of genes and for their potential treatment of human diseases. There are considerable excitements about its potential therapeutic applications in human diseases [1-3]. RNAi offers the prospects of higher specificity, lower immunogenicity, and greater disease modification than current antibody therapies for systemic autoimmune diseases (AID) such as systemic lupus erythematosus (SLE). The major challenge in turning RNAi into an effective therapeutic strategy is the delivery system.

JC virus (JCV), a human polyomavirus, belongs to the polyomaviridae. The JC virion contains three capsid proteins (VP1, VP2 and VP3) and a viral mini chromosome. VP1 is the major capsid protein constituting approximately 75% of the total proteins. Chang *et al. *[4] found that JCV VP1 could be transported into the nucleus and self-assembled to form capsid-like particles (VLPs) similar to the natural empty capsid without the involvement of the viral minor capsid proteins, VP2 and VP3. JCV VLPs can be generated by recombinant JCV VP1 protein in yeast expression. The recombinant VLPs were demonstrated to be able to package and deliver exogenous DNA into mammalian cell [5,6].

Patients with SLE produce large amounts of IL-10 in their serum which correlate with disease activity [7,8]. Administration of IL-10 antagonists has been found to be beneficial in the management of human SLE [9] or its murine [10,11]. The decrease in IL-10 level may contribute to the amelioration of the disease symptoms. Thus, IL-10 appears to play a key role in the autoimmune responses and might serve as a therapeutic target for SLE.

In this study, we show that JCV VLPs can be used as a gene delivery vector for IL-10 RNAi and for the possibility of gene therapy in SLE in the future.

## Methods

### Cell culture

The murine macrophage RAW 264.7 cell line was grown in 90% DMEM and 10% fetal bovine serum (FBS) obtained from Gibco BRL (Grand Island, NY) at a temperature of 37°C under a humidified and 5% CO_2 _atmosphere.

### Cell viability

Cells were counted using the trypan blue exclusion assay. The extent of cell viability was calculated and the viable cell numbers from experiment groups were compared with those in the untreated control groups.

### JC virus (JCV) virus-like particles (JCV VLPs)

JCV VLPs were generated by recombinant JCV VP1 protein in yeast expression system [12]. VLPs were further purified by sucrose gradient (10-30%) centrifugation for 40 min at 35,000 rpm. Particle-containing fractions were analyzed by hemagglutination activity after dialyzis in Tris buffer. VLPs were concentrated by ultracentrifugation for 3 h at 35,000 rpm and resuspended in 100 μl PBS.

### Construction of shRNA templates of IL-10

Two target sites were selected from mouse IL-10 (**NM_000572**) cDNA for generating two short hairpin RNA (shRNA) templates. Sequences for the target sites are GCTTCCAAACTGGATATAA and GTCTTCTGGAGTTCCGTTT respectively. For each shRNA template, two oligonucleotides containing partial complementary sequence of the shRNA with an overlapping loop region were synthesized and annealed as a shRNA cassette. The shRNA cassette was inserted into pcDNA/HU6 vector and introduced into *E. coli*[13]. The HU6-shRNA DNA fragment was polymerase chain reactions (PCR) amplified by a set of primers on the vector of flanking the HU6-shRNA template. Oligonucleotides used for target site #1 (target site sequences are in bold). IL10-RNAi-1-Forward: 5'-GATCC**GCTTCCAAACTGGATATAA**TTCAAGAGAT; IL10-RNAi-1-Reverse: 5'-AGCTTAAAAAAGCTTCCAAACTGGATATAATCTCTTGAAT'; Oligonucleotides used for target site #2 (target site sequences are in bold); IL-10 RNAi-2-Forward: 5'-GATCC**GTCTTCTGGAGTTCCGTTT**TTCAAGAGAA; IL10-RNAi-2-Reverse: 5'-AGCTTAAAAAAGTCTTCTGGAGTTCCGTTTTCTCTTGAAA.

### Packaging shRNA into JCV VLPs

Briefly, 100 μg of purified JCV-VLPs were mixed with 1 μg of PCR amplified shRNA template in capsid buffer (150 mM NaCl, 10 mM Tris-HCl, 0.01 mM CaCl_2_) and was incubated for 10 min at 37°C. Osmotic shock was achieved by diluting the mixture with 350 μl of distilled water and incubated for 20 min at 37°C [14].

### VLPs with IL-10 shRNA template or pEGFP-N3 for RAW264.7 cells

RAW 264.7 cells were grown on cover slips in 35-mm dishes overnight with DMEM supplement with 10% fetal calf serum. Cells were washed with PBS and incubated with 10 μg of VLPs containing the shRNA or pEGFP-N3 for 1 h at 37°C. Cells were then washed with PBS three times. Complete DMEM was added to the culture and incubated at 37°C with 5% CO_2 _for 48 h [14].

### Real-time PCR

All studies were carried out in a designated PCR clean area. RNA was extracted from cells using a Trizol reagent (Invitrogen, Carlsbad, CA, USA) according to the manufacturer's instructions. Total RNA was isolated from the RAW 264.7 cells incubated with LPS or VLPs with IL-10 shRNA. RNA samples were resuspended in diethyl pyrocarbonate (DEPC)-treated water, quantified, and then stored at -80°C until used. RNA concentration and purity were determined by a spectrophotometer by calculating the ratio of optical density at wavelengths of 260 and 280 nm. The first-strand of cDNA for RT-PCR was synthesized from the total RNA (2 μg) using the Promega RT-PCR system (Promega, Madison, Wisconsin, USA). The cDNA was denatured for 10 min at 95°C. Specific DNA fragments were amplified with a Max3000p Stratagene cycler with 40 cycles of 15 s at 95°C, 60 s at 60°C and 30 s at 72°C. The oligonucleotide primers were as follows: for mouse IL-10, 5'-CACTACCAAAGCCACAAAGCA-3' (forward) and 5'-AGGAGTCGGTTAGCAGTATGTT-3' (reverse). The amount of amplified DNA fragments encoding IL-10 was normalized to that of fragments encoding 18 s rRNA. Results are presented as the 'relative expression' in gene expression.

### Animals

Six-week-old female BALB/c mice were obtained from the National Animal Center, Taipei, Taiwan. The mice were divided into four groups with the treatment of PBS, LPS, LPS-VLPs-irrelevant shRNA and LPS-VLPs-IL-10 shRNA. LPS was from *E*. *coli *(Sigma Chemical Co. st. Louis, MO, USA; serotype 0111: B4). The animal experiments were approved by the Animal Research Committee of Chung Shan Medical University, Taiwan.

### IL-10 shRNA in BALB/c mice

Groups of BLAB/c mice were intraperitoneally injected with a single dose of 25 μg of LPS in 100 μl PBS. After two hours, mice were introvenousely treated with 250 μg of JCV VLPs with IL-10 shRNA, or irrelevant shRNA, respectively. After 6, 12 and 24 h of treatments, serum samples were collected and tested for IL-10 and TNF-α levels by real-time PCR and cytokine enzyme-linked immunosorbent assay (ELISA).

### Cytokine enzyme-linked immunosorbent assay (ELISA)

Conditioned RAW 264.7 cells medium and mouse serum were collected for subsequent analysis of cytokines, respectively. The levels of IL-10 and TNF-α were analyzed by using cytokines specific ELISA kits (IBL Co. Ltd, Gunma, Japan).

### Statistical analysis

Statistical analysis was performed using the paired *t*-test. A statistically significant difference was considered to be present at *P *< 0.05.

## Results

### Purification of JCV virus-like particles (VLPs)

**Figure 1:**
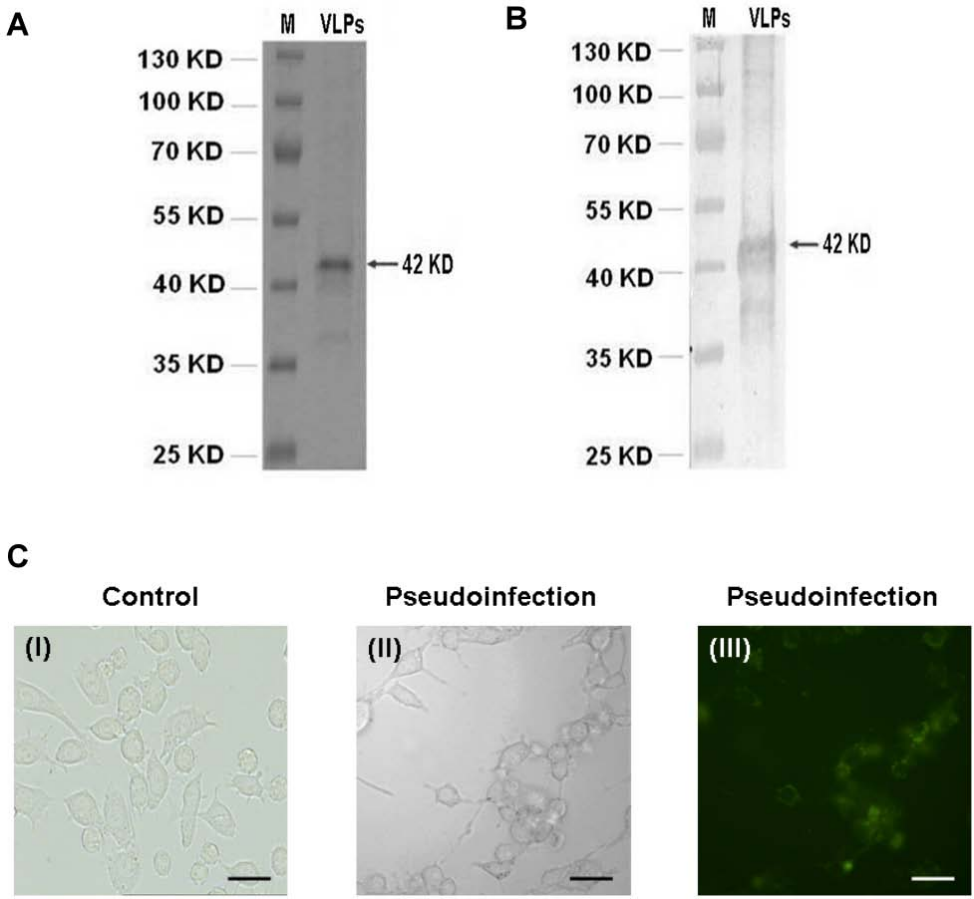
**Purification of JCV virus-like particles (VLPs)**. Recombinant JCV VLPs protein in yeast cells was purified and identified by SDS-PAGE (A) and Western blot (B). Lane M: molecular weight marker; Lane VLPs: JCV virus-like particles. The rabbit antibody reactive to the JC virus-like particle is indicated by an arrow. (C) RAW 264.7 cells were pseudo-infected with VLPs packaged with pEGFP-N3 (II, III) and were analyzed by light microscopy (II) and fluorescence microscopy (III). Light microscopy of RAW 264.7 cells with pseudo-infected with VLPs as a control (I).

Recombinant JCV VLPs protein in yeast cells was purified and identified by SDS-PAGE (Fig. 1A) and Western blot (Fig. 1B) using the rabbit antibody to JCV VLPs. The JCV VLPs showed strong hemagglutination activity and the activity was completely inhibited by JCV-positive human serum. As the control, RAW 264.7 cells were pseudoinfected with VLPs (Fig. 1C (I)), VLPs packaged with pEGFP-N3 were analyzed by light microscopy (Fig. 1C (II)) and fluorescence microscopy (Fig. 1C (III)). All RAW 264.7 cells transfected with VLPs expressed green fluorescence (Fig. 1C (III)).

### Suppression of IL-10 expression by IL-10 shRNA in RAW 264.7 cells

**Figure 2:**
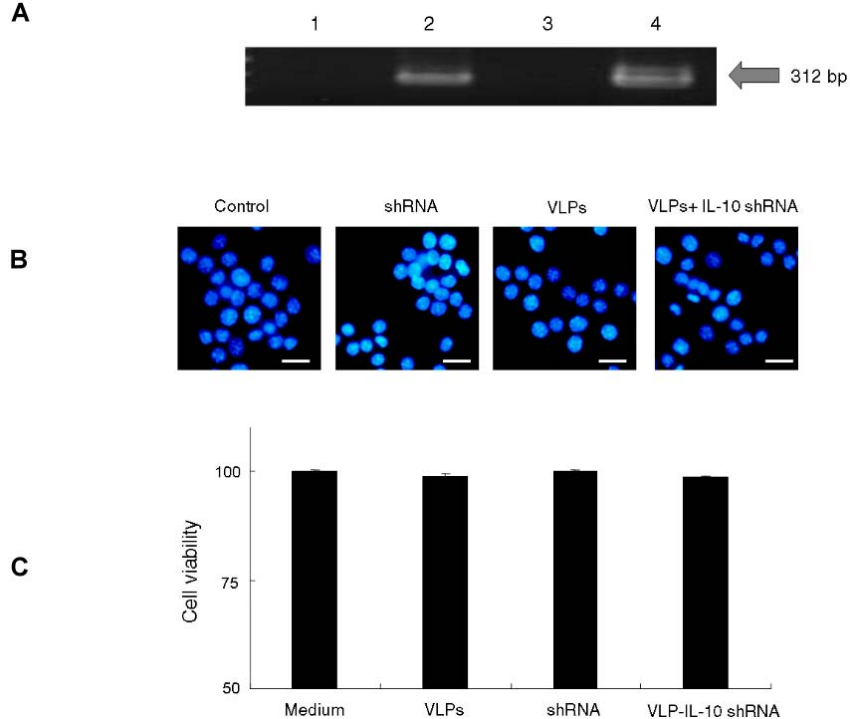
**Effects of VLPs IL-10 shRNA on morphology and viability of RAW 264.7 cells**. (A) Two preparations of IL-10 shRNA (IL-10i-1 and IL-10i-2) were packaged into JCV VLPs by osmotic shock. The presence of IL-10 shRNA template in VLPs was confirmed by PCR. Lane 1: VLPs; Lane 2: JCV VLPs with IL-10i-1 shRNA template; Lane 3: VLPs; Lane 4: VLPs with IL-10i-2 shRNA template. (B) RAW 264.7 cells were pseudo-infected with VLPs packaged with IL-10 shRNA and the effects of cell apoptosis were determined by 2-(4-Amidinophenyl)-6-indolecarbamidine dihydrochloride (DAPI) staining. (C) Cell viability was determined by trypan blue staining. VLPs IL-10 shRNA, VLPs only, or shRNA only did not induce cell death.

**Figure 3:**
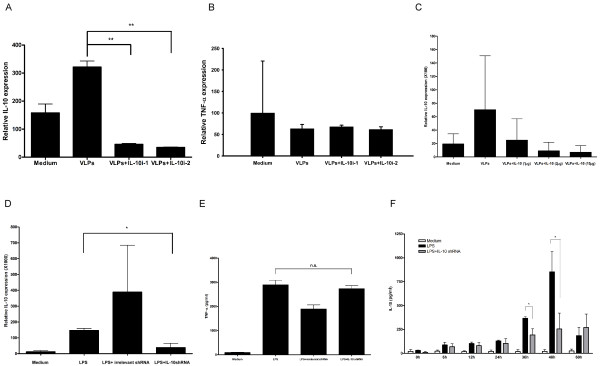
**Suppression of IL-10 expression in RAW 264.7 cells by two IL-10 shRNA preparations**. Cells were cultured with either IL-10 shRNA (IL-10i-1 or IL-10i-2) or irrelevant shRNA for 24 h. After washing, gene expression was analyzed by real-time PCR. Represented are the mRNA levels of target gene relative to those of 18 s rRNA. (A) Suppression of IL-10 expression was analyzed by real-time PCR. (B) IL-10 shRNA did not cross-react with TNF-α mRNA and influence the expression of TNF-α in RAW 264.7 cells. Relative TNF-α expression was analyzed by Real-time PCR. (C) Suppression of IL-10 expression by various doses of IL-10 shRNA in RAW 264.7 cells. (D) RAW 264.7 cells (2 × 10^5^) were pre-treated with LPS (2 μg/ml) for 90 min. Cells were then cultured with either IL-10 shRNA (IL-10i-2) or irrelevant shRNA for 24 h. After washing, IL-10 expression was analyzed by real-time PCR. Represented are the mRNA levels of IL-10 relative to those of 18 s rRNA. *: *P < 0.05*; ***: *P < 0.005*. (E) RAW 264.7 cells (2 × 10^5^) were pre-treated with LPS (2 μg/ml) for 90 min. Cells were then cultured with either IL-10 shRNA (IL-10i-2) or irrelevant shRNA for 36 h. After 36 h, conditioned medium was collected for analysis of TNF-α expression by ELISA. (F) RAW 264.7 cells (2 × 10^5^) were pre-treated with LPS (2 μg/ml) for 90 min. Cells were then cultured with either IL-10 shRNA (IL-10i-2) or irrelevant shRNA for 0, 6, 12, 24, 36, 48 and 60 hours. After 0, 6, 12, 24, 36, 48 and 60 hours, conditioned medium was collected for analysis of IL-10 expression by ELISA. *: *P < 0.05*.

Two preparations of IL-10 shRNA (IL-10i-1 and IL-10i-2) were packaged into JCV VLPs by osmotic shock and were confirmed by PCR (Fig. 2A). The morphology of RAW 264.7 cells with pseudoinfection of VLPs-IL-10 shRNA was shown in Fig. 2B. The cells with the pseudoinfection of VLPs were stained with DAPI (Fig. 2B) and the cell viability was detected by trypan blue staining (Fig. 2C). Figure 3 shows suppression of IL-10 expression in RAW 264.7 cells. Both IL-10i-1 and IL-10i-2 could reduce IL-10 expression about 85% and 89%, respectively, by real-time PCR compared to VLPs only (Fig. 3A) (*p < 0.005*). The expression of TNF-α was not affected by IL-10 shRNA treatment (Fig. 3B). The suppression of IL-10 by IL-10 shRNA was dose-dependent (Fig. 3C). Figure 3(D) shows the suppression of IL-10 by IL-10 shRNA after LPS stimulation in RAW 264.7 by real-time PCR. The irrelevant shRNA did not suppress the production of IL-10. The IL-10 shRNA packaged in JCV VLPs could sufficiently suppress IL-10 expression in RAW 264.7 cells. We also found that the expression of TNF-α was not affected by IL-10 shRNA after LPS treatment in RAW264.7 cells by ELISA (Fig. 3E). Figure 3F shows reduction of IL-10 by IL-10 shRNA after LPS treatment in RAW 264.7 cells at 36 and 48 hours by ELISA. In addition, the IL-10 suppressive effects could be sustained for 48 h by IL-10 shRNA. Thus, we found suppression of IL-10 by VLPs-IL-10 shRNA not only at mRNA levels but also at protein level.

### Effects of IL-10 shRNA on cytokines production of IL-10 and TNF-α in BALB/c mice

**Figure 4:**
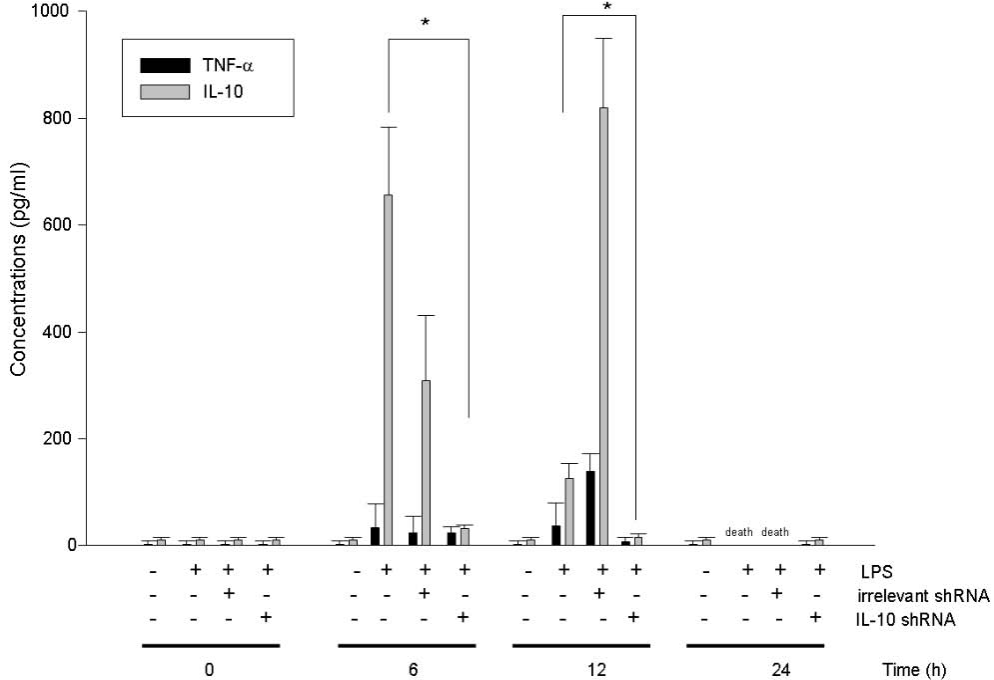
***In vivo *effects of IL-10 shRNA on production of IL-10 and TNF-α in BALB/c mice**. Groups of BALB/c mice were intraperitoneally injected with LPS (25 μg). After two hours, mice received VLPs-IL-10 shRNA or irrelevant shRNA intravenously. After 6, 12, 24 and 36 h, serum samples were collected from each mouse and tested for IL-10 and TNF-α levels by ELISA. The level of IL-10 was suppressed after the treatment of IL-10 shRNA. *: *P < 0.05*; ***: *P < 0.005*.

After LPS treatment in mice, IL-10 was increased and the peak concentration of IL-10 was at 6 h then decreased at 12 and 24 h. In the group of mice with LPS-irrelevant shRNA treatment, IL-10 was also increased at 6 h, but the peak concentration was delayed to 12 h. The concentration of IL-10 was higher in the group of mice treated with LPS-irrelevant shRNA than these with LPS only treatment (Fig. 4). The irrelevant shRNA itself could obviously induce IL-10 production. The production of IL-10 was decreased after VLPs with IL-10 shRNA treatment (93% of suppression) (*P < 0.05*). There was no suppression of IL-10 in the control groups treated with PBS, LPS and LPS-VLP-irrelevant shRNA.

### Effects of TNF-α

TNF-α was also increased after LPS injection and the peak concentration of TNF-α was at 24 h. TNF-α was increased in the groups with LPS and LPS-irrelevant shRNA treatment. The concentration of TNF-α was decreased after JCV VLPs IL-10 shRNA treatment (81% of suppression) (Fig. 4). IL-10 shRNA did not cross-react with TNF-α mRNA and influence the expression of TNF-α in RAW 264.7 cells (Fig. 3B). In BALB/c mice, however, down regulation of IL-10 by IL10 shRNA could affect TNF-α expression (Fig. 4).

### Effects on survival

**Figure 5:**
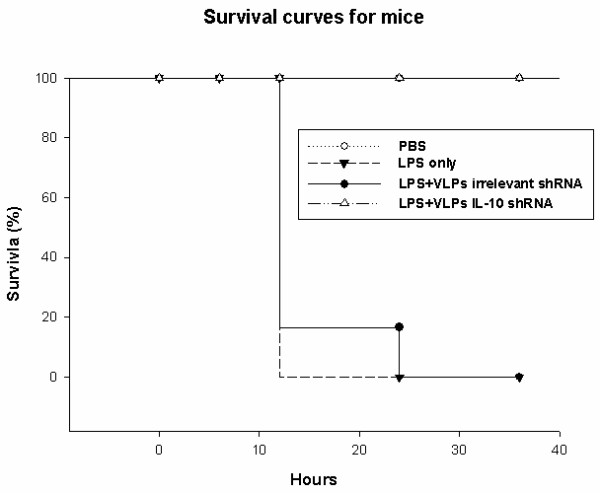
**Outcome of BALB/c mice treated with LPS, irrelevant shRNA and IL-10 shRNA**. Groups of BALB/c mice were intraperitoneally injected with LPS (25 μg). After two hours, mice received intravenously VLPs-IL-10 shRNA and irrelevant shRNA. The survival rate and survival time of the animals were observed and compared between groups.

All mice treated with PBS only or LPS-VLPs-IL-10 shRNA survived in the end of the experiment, but all mice treated with LPS only or LPS-VLPs irrelevant shRNA died after 24 h of treatment. The mice treated with LPS-VLPs-IL-10 shRNA had longer lifespan than those treated with LPS-VLPs irrelevant shRNA or LPS. (Fig. 5)

## Discussion

We have shown for the first time that JCV VLPs could be used as a vector to deliver IL-10 shRNA in both RAW 264.7 cells and BALB/c mice. The JCV VLPs with IL-10 shRNA could effectively silence the IL-10 cytokine both *in vitro *and *in vivo *and were not toxic to RAW 264.7 cells. These findings suggest that JCV VLPs is an effective delivery vector for RNAi delivering with potentially therapeutic use for autoimmune diseases such as SLE.

Among methods for gene transfer into cells, viral gene-delivery system is a most effective method. In previous studies, we demonstrated that the recombinant JC virus major capsid protein, VP1, is able to self-assemble to form a virus-like particle (VLP) when expressed in the baculovirus [4], *E. coli *[14] and yeast [12]. In addition, the VLP is able to package exogenous DNA and deliver it into human kidney [14], glioma [5] and colon adenocarcinoma [15] cells. These findings indicate that the JCV VLPs may be used as a gene delivery vector for therapeutic applications. In addition, JCV VLPs may be used for human therapy [15]. In this study, we further extended the findings that JCV VLPs provided an efficient tool for delivering shRNA to silence the targeted RNA. Because of lack of viral nucleic acids in JCV VLPs, JCV do not cause serious risks for infection and are considered as a safe and efficient method for transfection of RNAi. The JCV receptor is widely distributed among mammalian cells including human, monkey, and mouse. JCV can enter a wide variety of cell types [16]. However, the immune response is a limitation when developing a viral gene delivery vectors. For human polyomaviruses, most adults are seropositive against JCV. It may not beneficial to use the JCV VLPs as a gene delivery vector in such circumstances. Therefore, modification of the JCV VLPs to avoid immune elimination may be useful in advancing the development of the JCV as a gene delivery vector in human.

We used the HU6-shRNA template for expressing RNAi. Since VLPs has a certain DNA packing capacity, to increase the ratio of packed shRNA template, a minimal linear DNA fragment containing HU6 promoter and shRNA template (HU6-shRNA template) was PCR amplified and packed into VLPs. This linear HU6-shRNA template was efficiently packed into VLPs and expressed successfully in cells and animals. Other than more economic, another advantage of using HU6-shRNA template is that the shRNA can be synthesized continuously as long as the DNA template exists, while siRNA will be cleaved along with target mRNA degradation.

In this study, we used two IL-10 shRNAs which target different sites of IL-10 in RAW264.7 cells and found that both IL-10 shRNAs efficiently reduced IL-10 mRNA but not TNF-α mRNA level (Fig 3. B). We have two reasons to believe that there is no cross reaction between IL-10 shRNA and TNF-α mRNA. First, both IL-10 shRNAs did not affect TNF-α mRNA level. Second, since RNAi is mainly functioning on mRNA level of target gene and real-time PCR is a sensitive method to quantitate the level of mRNA, we are confident that IL-10 shRNA did not react with TNF-α mRNA. However, down regulation of IL-10 via shRNA is accompanied by reduction of TNF-α level in an animal model (Fig. 4). This could be explained by the possibility that down regulation of IL-10 may affect TNF-α expression in other cell types through paracrine system.

Toxic shock is mediated by a complex set of cytokine interactions. Previous studies showed that infusion of LPS mimics the endotoxic state and results in the production of multiple inflammatory cytokines, including IL-1, INF-γ and TNF-α production, which is known to cause tissue necrosis and organ failure. In addition to inducing the production of pro-inflammatory cytokines, LPS also triggers the release of anti-inflammatory cytokines, such as IL-10, in the bloodstream of normal mice [17]. Many cell types can produce IL-10, including phagocytic cells, conventional DCs, T cells, B cells, and NK cells. IL-10 has been implicated as a key anti-inflammatory modulator in the cascade of cytokine synthesis, and was identified as a critical counterbalance to proinflammatory cytokine synthesis. It acts to terminate the inflammatory response and limit inflammation-induced tissue pathology by deactivating macrophages and silencing their synthesis of TNF-α, IL-6, IL-1α, IL-8, and an array of other proinflammatory cytokines and chemokines. In mouse models of toxic shock, IL-10 is produced very rapidly after exposure to LPS [18,19] and serum levels of INF-γ and IL-10 peak at the same time as TNF-α, which is known to play a central role in the pathogenesis of toxic shock. In such models, it was observed that IL-10 appears in the serum within one hour after LPS challenge and persists in the blood for at least six hours [18,19]. Subsequently, it was found that a single injection of IL-10 prevented death in murine models of LPS-induced toxic shock, and maximum protection was afforded only when IL-10 was given shortly before or after IL-10 challenge [18,20]. Neutralization of endogenously produced IL-10 by administration of anti-IL-10 monoclonal antibody 2 h before LPS challenge resulted in a marked increase in both TNF-α and IFN-γ serum levels, and high mortality rates [19], suggesting that the rapid release of IL-10 during endotoxemia is a natural antiinflammatory response controlling cytokine production and LPS toxicity. Increased lethality was also observed when anti-IL-10 antibody treatment was given at the same time as LPS [19,21], but not when the antibody treatment was delayed until 3 hours after challenge [21], suggesting that IL-10 mediates protection in the earliest phase of the LPS response. In the current study, the anti-IL-10 shRNA was administered to mice two hours after LPS injection. This could explain, only in part, the beneficial effects on survival we have observed.

Another potential explanation stems from the complexity of functions played by IL-10 in the immune system. Although IL-10 is considered a potent antiinflammatory cytokine, studies have suggested that IL-10 also possesses immunostimulatory effects [22,23]. When administered 1 h after LPS injection, it potentiated LPS-induced IFN-release, which was associated with elevated levels of the IFN-dependent chemokines. In addition, IL-10 treatment enhanced activation of CTL and NK cells after LPS injection. Since, under certain conditions, IL-10 has proinflammatory functions in vivo, stimulating CD4+, CD8+ T cells, and/or NK cells, it is possible that the anti-IL-10 shRNA used in the current work blocked profoundly IL-10 production in several cell types, leading to less inflammation and lower mortality rates in mice exposed to toxic shock.

In this study, BALB/c mice treated with IL-10-VLPs-shRNA decreased production of IL-10 (93% suppression). This finding provides a good model using JCV VLPs as a delivery tool for RNAi *in vivo*. IL-10 is a key regulator of the immune system and has been reported to be associated the development of SLE. The decrease in the expression level of IL-10 may contribute to the amelioration of the disease symptoms in human SLE [9]. It has been reported that treatment with anti-IL-10 antibody delayed onset of autoimmunity in (NZW/NZB)F1 mice and led to a reduction in disease activity [10,11]. RNAi offers the prospects of higher specificity, lower immunogenicity, and greater disease modification than current antibody therapies.

In conclusion, JCV VLPs is an easily prepared agent capable of effectively delivering RNAi *in vitro *and *in vivo *without significant cytotoxicity. The system provides an efficient tool for delivering the RNAi for the possibility of gene therapy in the future.

## Competing interests

The authors declare that they have no competing interests.

## Authors' contributions

MIC and YFH contributed equally to this work. MIC and YFH interpreted the data and drafted the manuscript. MIC, MW, DC and JTC designed and performed the RNAi and JC virus experiments. MZ and GJT conceived of this study, and participated in its design and coordination. All authors read and approved the final manuscript.
